# *Bacillus amyloliquefaciens* MBI600 differentially induces tomato defense signaling pathways depending on plant part and dose of application

**DOI:** 10.1038/s41598-019-55645-2

**Published:** 2019-12-13

**Authors:** Anastasia Dimopoulou, Ioannis Theologidis, Burghard Liebmann, Kriton Kalantidis, Nikon Vassilakos, Nicholas Skandalis

**Affiliations:** 1 University of Crete, Department of Biology, 70013 Heraklion, Greece; 2 Institute of Molecular Biology and Biotechnology, FORTH, 100N. Plastira str, 70013 Heraklion, Greece; 3 Global R&D Biologicals, BASF SE, 67117 Limburgerhof, Germany; 4 Keck School of Medicine of University of Southern California, HSC 1441 Eastlake Ave, Los Angeles, 90033 CA USA

**Keywords:** Antimicrobials, Bacteria, Plant sciences, Plant hormones, Plant molecular biology, Plant signalling, Plant stress responses

## Abstract

The success of *Bacillus amyloliquefaciens* as a biological control agent relies on its ability to outgrow plant pathogens. It is also thought to interact with its plant host by inducing systemic resistance. In this study, the ability of *B*. *amyloliquefaciens* MBI600 to elicit defense (or other) responses in tomato seedlings and plants was assessed upon the expression of marker genes and transcriptomic analysis. Spray application of Serifel, a commercial formulation of MBI600, induced responses in a dose-dependent manner. Low dosage primed plant defense by activation of SA-responsive genes. Suggested dosage induced defense by mediating synergistic cross-talk between JA/ET and SA-signaling. Saturation of tomato roots or leaves with MBI600 elicitors activated JA/ET signaling at the expense of SA-mediated responses. The complex signaling network that is implicated in MBI600-tomato seedling interactions was mapped. MBI600 and flg22 (a bacterial flagellin peptide) elicitors induced, in a similar manner, biotic and abiotic stress responses by the coordinated activation of genes involved in JA/ET biosynthesis as well as hormone and redox signaling. This is the first study to suggest the activation of plant defense following the application of a commercial microbial formulation under conditions of greenhouse crop production.

## Introduction

*Bacillus amyloliquefaciens* strains are used commercially as biological control agents (BCAs) that suppress plant pathogens^1^. Their mode of action involves synthesis and secretion of secondary metabolites with antibiotic function; also competition for nutrients and root colonization^2^. Proliferation on root surfaces prevents the entry of phytopathogens but also initiates interactions with the plant host that trigger defense mechanisms^3^.

It is known that plants respond to attack by inducing two distinct defense mechanisms, the systemic acquired resistance (SAR) and the induced systemic resistance (ISR)^4–6^. Both are effective against multiple but different types of pathogens^7^. The two defense mechanisms use different pathways of plant metabolism; SAR requires the synthesis of salicylic acid (SA), which in turn triggers the expression of a well-known set of Pathogen-Related (PR) genes^8,9^, while ISR is dependent on jasmonic acid (JA) and ethylene (ET) signaling pathways^10,11^. The interplay between SA and JA responsive pathways involves synergism or antagonism which allows plant hosts to prioritize them, depending on the sequence and type of attackers encountered^12–14^. A major constituent of this hormonal networking is ET that can act both positively and negatively on plant immunity, by suppressing SA-responsive signaling pathways while activating their JA-responsive counterparts and vice versa^15^. Apart from these three major defense hormones, abscisic acid (ABA), auxin, gibberellins (GAs) and cytokinins also function, in synergy or antagonism, as secondary regulators of the plant immunity network^16–19^.

Since the early 1990s, activation of ISR by plant growth-promoting rhizobacteria (PGPR) has been investigated as a possible practical way to use induced resistance in agriculture^20^. *Arabidopsis thaliana* plants, when treated with PGPR are thought to respond with the induction of ISR via the JA/ET – NPR1 dependent signaling pathway while SA is gradually suppressed by this synergistic action^21^. In support, *Bacillus amyloliquefaciens* FZB42 was able to enhance the expression of defense marker genes such as *pr1* (SA marker gene) and *pdf1*.*2* (JA/ET marker gene) in lettuce plants exposed only to FZB42, while the simultaneous presence of FZB42 and the lettuce pathogen *Rhizoctonia solani* still activated *pdf1*.*2* but *pr1* expression levels were lower compared to the FZB42 treatment^22^. It was suggested that both SA and ET signaling pathways are responsive, at first, to the synergistic activation of JA/ET signaling pathways, of which SA signaling is suppressed upon pathogen attack. Inoculation of *Arabidopsis* plantlets with *Bacillus subtilis* FB17 also induced *pdf1*.*2* and *pr1* transcript levels^23^. Despite the accumulating knowledge on PGPR-mediated induction of resistance responses, little is known about the ability of commercial formulations of BCAs (containing dormant cells or endospores) to elicit plant defenses following root or leaf treatment. Moreover, the signaling network that is involved at the BCA-mediated induction of defense responses in real crop production conditions remain to be elucidated.

In this study, based on previous findings suggesting induction of tomato resistance to viral pathogens by *B*. *amyloliquefaciens* subsp. *plantarum* MBI600^24^, we thoroughly investigated the activation of tomato defense mechanisms by Serifel, a commercial formulation containing MBI600 endospores, with confirmed fungicidal and bactericidal functions. The induction and interplay of ISR and SAR, activated by the presence of MBI600 in tomato rhizosphere or phyllosphere was predicted based on the up-regulation of a total of 8 salicylic acid, jasmonic acid or ethylene-responsive marker genes. Activation was assessed, using real-time qPCR, at 10 time points following drench or spray application of 4-week-old tomato plants. The use of increasing concentrations of MBI600 in application schemes revealed that synergy or antagonism between ISR and SAR signaling pathways depends on the abundance of microbial elicitors perceived. To confirm this, we saturated the leaf and root surface with such elicitors, in the form of an MBI600 cell-free culture filtrate (CFCF). The transcriptome of tomato seedlings treated with CFCF was analyzed and significant differentiation of regulation of 24 genes was confirmed using qRT-PCR. Some of these genes are identified for the first time in PGPR-plant interactions. A pathway analysis of early and late responses allowed the suggestion of a signaling network that is triggered by MBI600-tomato seedling interactions.

## Results

### Seedling root treatment with MBI600 elicitors activates ISR

Expression analysis of a series of ISR or SAR marker genes following drench application of Serifel on tomato plants suggested a mild effect on the activation of immune responses (Supplementary Fig. S1). Results exhibited an early response of both *pr1b1* and *loxD* transcription levels at 3 days post application (dpa) compared to control treatment and a significant up-regulation of *loxD* transcription levels at 10 dpa. No significant differences were observed among treatments on the expression levels of the rest of the genes.

**Figure 1:**
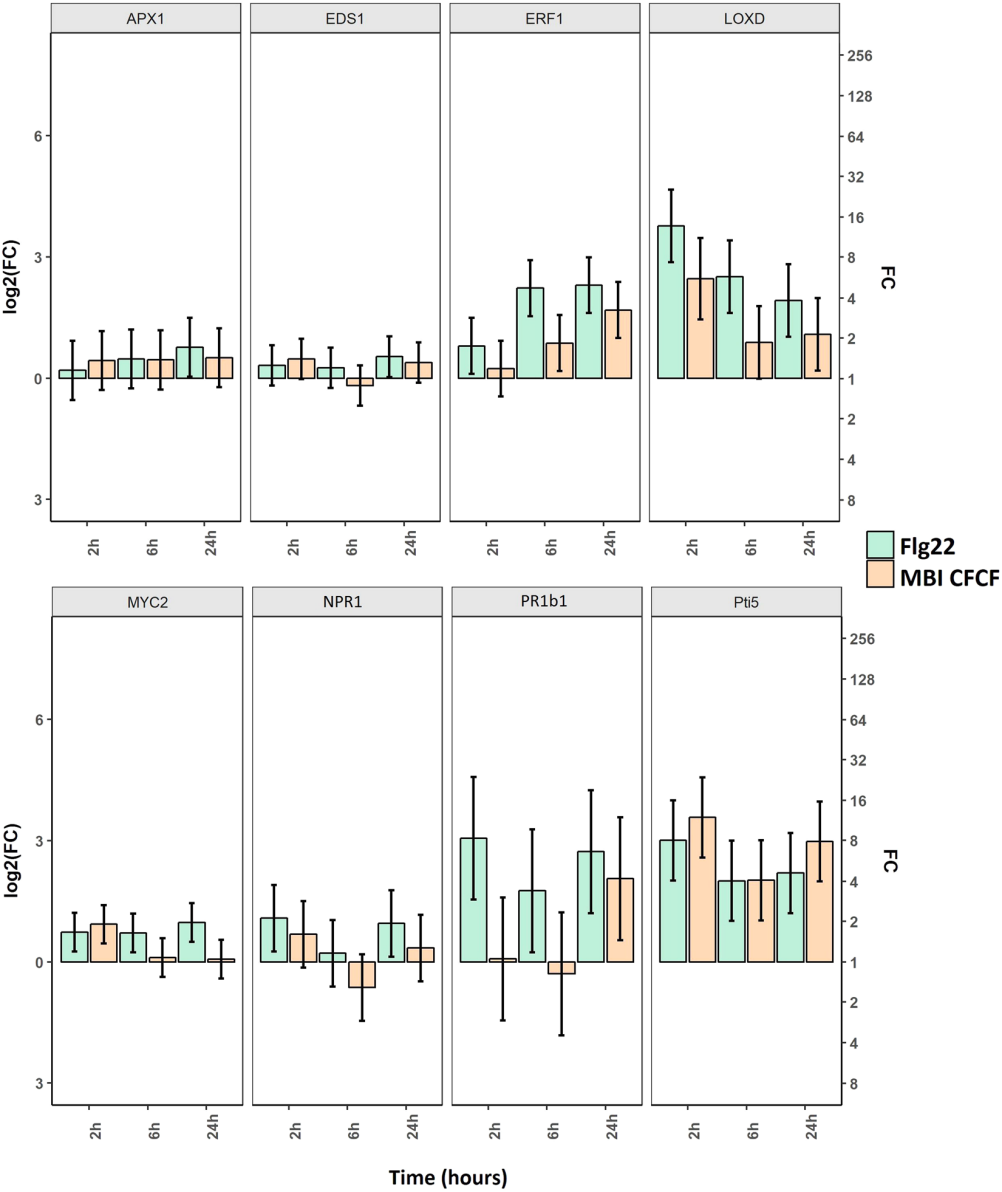
Expression levels of selected defense genes (*apx1*, ascorbate peroxidase 1; *eds1*, enhanced diseased susceptibility 1; *erf1*, ethylene response factor 1; *loxD*, lipoxygenase D; *myc2* transcription factor; *npr1*, non-expressor of PR 1; *pr1b1*, pathogenesis-related leaf protein 6 and *Pti5*, Pto-interacting protein 5) in response to MBI600 CFCF (or flg22) root application on tomato seedlings. Fold change (FC) represents the relative difference in expression between each treatment and control (LB + MS). Triplicates of pools of two individual seedlings were analyzed and the results of three combined experiments are presented. *UBI3* was used as a reference gene. Bars indicate the 95% confidence interval (2*SE). Estimates whose confidence interval includes the baseline value 0 (1 in fold-change scale) are not significantly different from control at α = 5%.

A similar analysis was performed following drench application of seedlings with MBI600 CFCF to maximize root contact with MBI600 elicitors; flg22 and water-LB treatment served as positive and negative controls, respectively (see Methods). Results (Fig. 1, Supplementary Table S2) showed that *erf1* transcripts gradually increased over time in both cases of MBI600 CFCF and flg22 compared to negative control whereas *loxD* transcripts peaked at 2 hours post application (hpa) and were then gradually decreased but remained in higher levels than those of negative control treatment at all time points. Expression trends were similar in the case of flg22. The CFCF effect on *loxD* expression was comparable to that of the flg22. Similarly, *pti5* transcript levels increased early after CFCF application (2 h) and remained elevated at 6 and 24 hpa, compared to negative control. Up-regulation of *pr1b1* transcriptional levels occurred late (24 hpa) in the case of CFCF and early in the case of flg22, compared to negative control. Expression levels of *myc2* were significantly increased early (2 h) after CFCF application, while *npr1* showed the same trend at 2 hpa, but comparisons were not statistically significant. No significant differences were observed among treatments in the expression levels of *apx1* and *eds1* genes.

**Figure 2:**
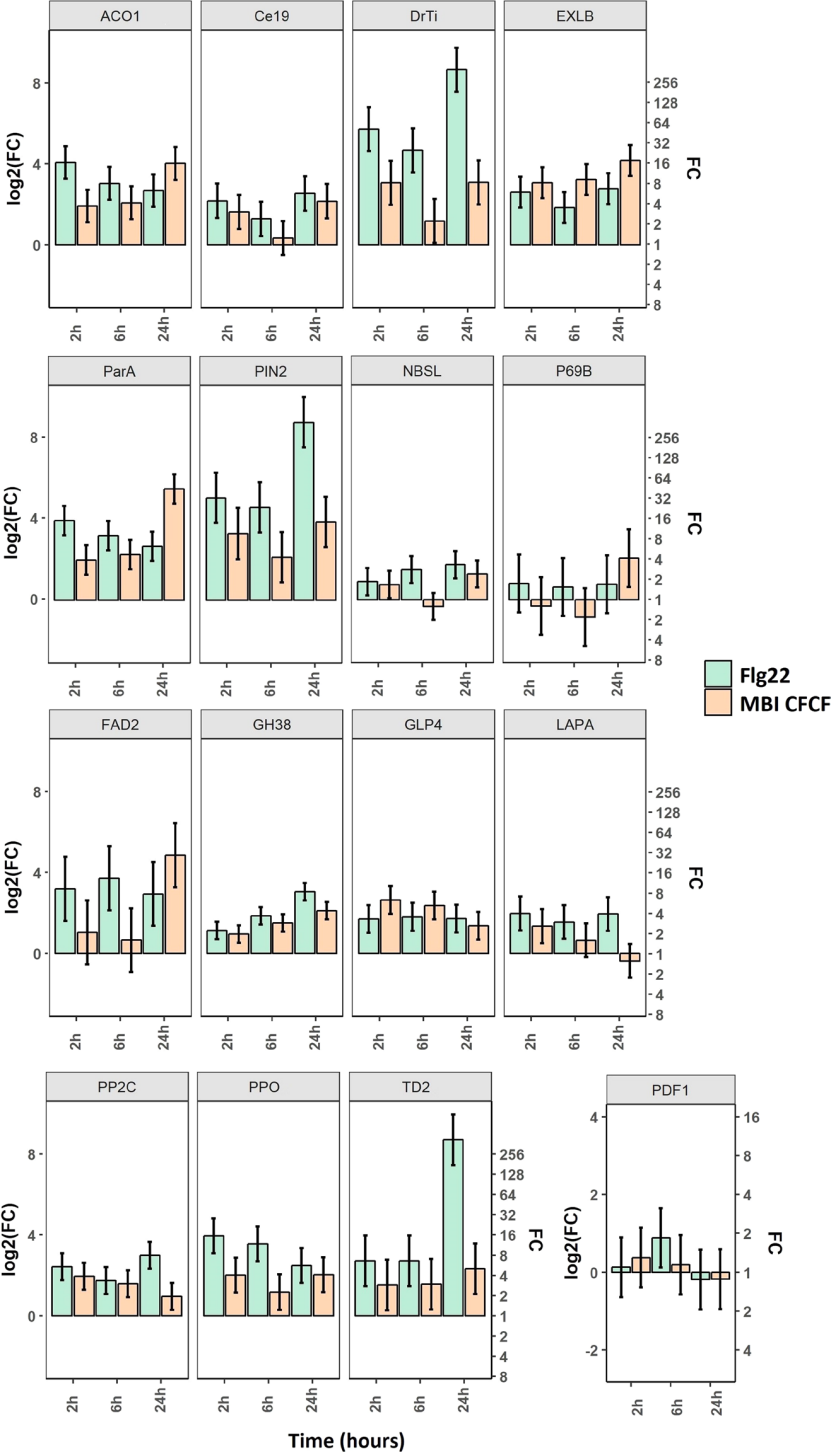
Expression levels of 16 genes (Supplementary Table S1) related to biotic and abiotic responses of tomato seedlings following root treatment drench with either MBI600 CFCF or flg22 1 μM. Triplicates of pools of two individual seedlings collected at 2, 6 and 24-hours post-treatment were analyzed and the results of three combined experiments are presented. Fold change (FC) represents the relative difference in expression between each treatment and control (LB + MS). *UBI3* was used as a reference gene. Bars indicate the 95% confidence interval (2*SE). Estimates whose confidence interval includes the baseline value 0 (1 in fold-change scale) are not significantly different from control at α = 5%.

For further investigation of interactions that occurred between MBI600 elicitors and roots of tomato plants, a transcriptomic analysis was performed on the same seedling samples. Each sample (flg22_6h or flg22_24 h, MBI_6h or MBI_24h, control_6h or control_24h), yielded at least 100 genes that were differentially expressed and selected based on P. values of comparisons between treatments and controls (https://www.ncbi.nlm.nih.gov/geo/query/acc.cgi?acc=GSE123589). The 74% or 57% of these genes were up-regulated, in the case of MBI_6h or MBI_24h samples. Respective numbers were 95% and 65% in the case of flg22_6h or flg22_24h (Supplementary Fig. S2). This analysis was used as a guideline for the selection of 16 differentially expressed genes (further to the 8 markers), favoring up-regulated genes that are implicated in hormonal responsive signaling or biosynthesis. Consistent with the microarray results, the expression analysis of all 16 genes showed elevated transcription levels following both MBI600 CFCF or flg22 treatments, in comparison to control treatment 6 and 24h (Fig. 2, Supplementary Table S2). In detail, transcriptional levels of *cevi19*, *DrTi*, *Pin2*, *NBS*-*LRR* and *ppo* exhibited a concave uptrend with peak values at 2 and 24 h while minimums were observed at 6 h after MBI600 CFCF application. All peaks at 2 and 24 h were statistically different from negative controls. The same trend was observed for flg22 in higher levels than those of ΜΒΙ600 CFCF, except for *ppo* transcripts that peaked at 2 h and were then gradually reduced. A significant up-regulation was observed for *p69b* and *fad2* expression at 24 h after CFCF treatment while no differences were observed at 2 and 6 hpa compared to negative controls. Interestingly, *fad2* transcripts in flg22 samples were higher than those of the negative control at all time points counter to *p69b* where no differences were observed. Transcript levels of *aco1*, *exlb1*, *parA*, *gh3*.8, and *td2* genes were gradually increased over time reaching a peak at 24 h after MBI600 CFCF application. Flg22 reduced the expression of *aco1* and *parA* over time but at all time points the transcription levels were higher than those of the negative control. Moreover, expression of *glp4*, *lapA* and *pp2c* was peaked at 2 hpa; all comparisons to controls were significant. Expression was then gradually decreased and only *glp4* and *pp2c* transcript levels still remained higher than those of respective controls at 6 and 24 hpa. Finally, *pdf1*.2 expression showed no differences among treatments except for flg22 treated sample at 6 hpa, in which case transcripts were elevated.

### MBI600 elicitors triggered immune responses in leaf tissue

**Figure 3:**
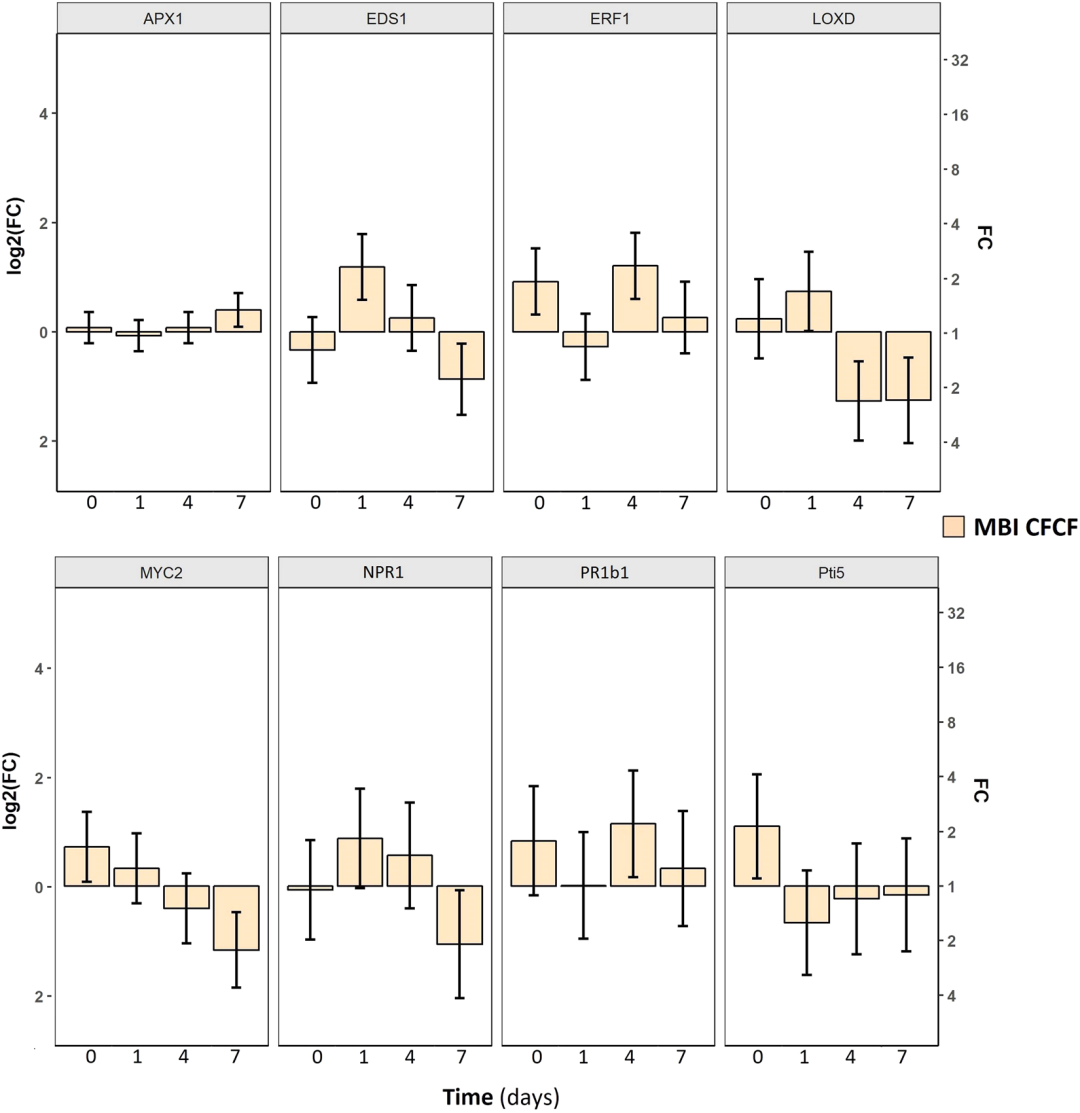
Effect of MBI600 CFCF foliar application on the transcriptional levels of selected defense genes (*apx1*, *eds1*, *erf1*, *loxD*, *myc2*, *npr1*, *pr1b1*, and *pti5*) in a duration of 7 days post application. Pools of four individual plants were analyzed and the results of two combined experiments are presented. Fold change (FC) represents the relative difference in expression between each treatment and water. *UBI3* was used as a reference gene. Bars indicate the 95% confidence interval (2*SE). Estimates whose confidence interval includes the baseline value 0 (1 in fold-change scale) are not significantly different from control at α = 5%.

MBI600 elicitor perception by leaf receptors was also investigated following CFCF spray application of tomato plants. Expression analysis showed elevated *erf1*, *myc2* and *pti5* expression instantly (0 dpa) after MBI600 CFCF treatment (Fig. 3, Supplementary Table S2). At 1 dpa, only *eds1* and *loxD* transcripts were significantly higher than water treatment, whereas the same response was recorded for *npr1* transcripts but differences to water treatment were marginal. Late responses were observed at 4 dpa for *erf1* and *pr1b1* transcriptional levels which were significantly higher, reaching approximately a 2-fold increase compared to water treatment, in both cases. In contrast, *loxD* transcripts were significantly lower. Interestingly, at 7 dpa transcriptional levels of *eds1*, *loxD*, *myc2* and *npr1* genes were significantly lower, while *erf1*, *pr1b1*, and *pti5* showed no significant differences compared to water treatment. Finally, *apx1* transcripts were elevated only at 7 dpa.

### Serifel activated immune responses in a dose-dependent manner

**Figure 4:**
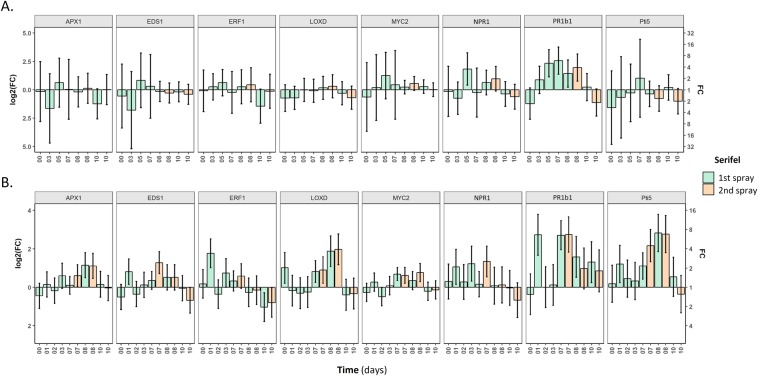
Expression levels of selected defense genes in response to Serifel foliar application. 4 weeks-old plants were treated twice by spraying with the. (**A**) Low dosage concentration 0.03 g/L. of Serifel or. (**B**) Suggested dosage concentration 1.25 g/L. in a 7-day interval. Pools of four individual plants were analyzed and the results of three combined experiments are presented. Fold change (FC) represents the relative difference in expression between each treatment and water. *UBI3* was used as a reference gene. Bars indicate the 95% confidence interval (2*SE). Estimates whose confidence interval includes the baseline value 0 (1 in fold-change scale) are not significantly different from control at α = 5%.

Activation of marker genes of ISR and/or SAR following Serifel spray application was monitored based on qRT-PCR expression analysis (Fig. 4, Supplementary Table S2). Suggested versus low dosages and one versus two application schemes were compared for such effects. The low spraying dosage was equal to that suggested for drench application in order to compare root with leaf effects.

Application of low dosage of Serifel had a significant effect only on *npr1* and *pr1b1* expression levels at 5 dpa compared to control (Fig. 4A). While *npr1* expression was decreased over time, *pr1b1* expression was sustained at high levels until 8 dpa, with a 6-fold peak at 7 dpa. On the contrary, most marker genes were activated following application of Serifel at the suggested dosage (Fig. 4B). In detail, an early (1 dpa) and significant up-regulation was observed in the case of *eds1*, *erf1*, *npr1*, *pr1b1* and *pti5* genes which was followed by an instant (2 dpa) reduction in levels comparable to those of the control. A similar expression pattern occurred in the case of *loxD* immediately after application (0 dpa), while no early responses were observed in the case of *apx1* and *myc2*. At 3 dpa there was a significant up-regulation only for the *npr1* gene. At 7 dpa, expression of *loxD*, *myc2*, *pr1b1* and *pti5* was significantly up-regulated. Seven, 2.5 or 2-fold differences compared to control were calculated in the case of *pr1b1*, *loxD* or *myc2*, respectively. Expression levels of *loxD* and *pti5* remained high at 8 dpa while those of *pr1b1* and *myc2* were gradually reduced. A late response (8 dpa) was observed in the case of *apx1*. The only gene found activated at 10 dpa was *pr1b1*.

Concerning one versus two Serifel applications, expression levels of *eds1* and *npr1* were up-regulated instantly following the second application (7 dpa) in comparison to control and single application treatments (Fig. 4, Supplementary Table S2). In the case of *apx1*, transcripts remained at high levels at 8 dpa in contrast to *eds1* and *npr1* transcripts which were reduced. Finally, the two application schemes had a positive effect on *myc2* expression levels which remained at high levels compared to one application scheme and controls at 8 dpa. At 10 dpa, only *eds1* and *erf1* expression levels were significantly down-regulated; the former solely in the two application schemes, while the latter in both.

In addition to marker genes, the same leaf tissue was used to assess the activation of genes that were identified by seedling experiments but have not been previously associated with PGPR-plant interactions. *exlb1* was significantly down-regulated at 8 dpa while no significant differences in comparison to control were observed at the rest of the time points (Supplementary Fig. S3). *fad2l* demonstrated a marginal induction early (1 dpa) and a significant induction late (8 dpa) after Serifel application, when it reached a 6-fold increase compared to control. A mild activation of *gh3*.8 occurred following Serifel application but comparisons to control were not statistically significant at any time point. Transcription levels of *parA* were immediately elevated after Serifel application (0 dpa), reaching a peak at 1 dpa. The same trend was observed at 7 and 8 dpa. Finally, *pp2c* expression levels were down-regulated at 0 and 2 dpa, significantly up-regulated at 3 and 7 dpa, and down-regulated again at 10 dpa.

## Discussion

Local and systemic defense responses triggered by beneficial and parasitic microorganisms are controlled by a signaling network in which plant hormones play a dominant role^21^. In contrast to plant pathogens, beneficial microorganisms have been thought to trigger mild plant responses that do not overall affect the plant host transcriptome^25,26^ and contribute to priming rather than the actual induction of systemic defense^27^. This study investigated the ability of Serifel, a commercial formulation containing pure *B*. *amyloliquefaciens* MBI600 endospores (BASF SE), to activate stress signaling responses of tomato plants. Moreover, Serifel activated defense mechanisms in a dose-dependent manner: *npr1* and *pr1b1* genes were up-regulated by low exposure to BCAs, indicative of a mild activation of defense mechanisms whereas, most genes were activated when BCA cues exceed a certain threshold. Accordingly, Serifel drench application at a dosage equal to low spray application up-regulated only *loxd* gene while MBI600 CFCF drench application in seedlings, which maximized host perception of MBI600 elicitors, activated most genes, suggesting an induction of defense mechanisms.

Serifel spraying at a low dosage activated only a few SA-responsive genes (*npr1*, *pr1b1*) whereas use of suggested dosage resulted in the activation of all JA/ET-responsive genes assessed (*erf1*, *loxD*, *myc2*), while the SA signaling pathway was still active (*eds1*, *npr1*, *pr1b1*; Fig. 4). This suggests a synergy between SA and JA/ET – responsive pathways, which was confirmed in experiments using MBI600 CFCF. Moreover, the second Serifel application elevated the expression of SA-responsive genes, suggesting a priming effect on SAR rather than ISR responses. We have recently reporteda similar observation^24^.

**Figure 5:**
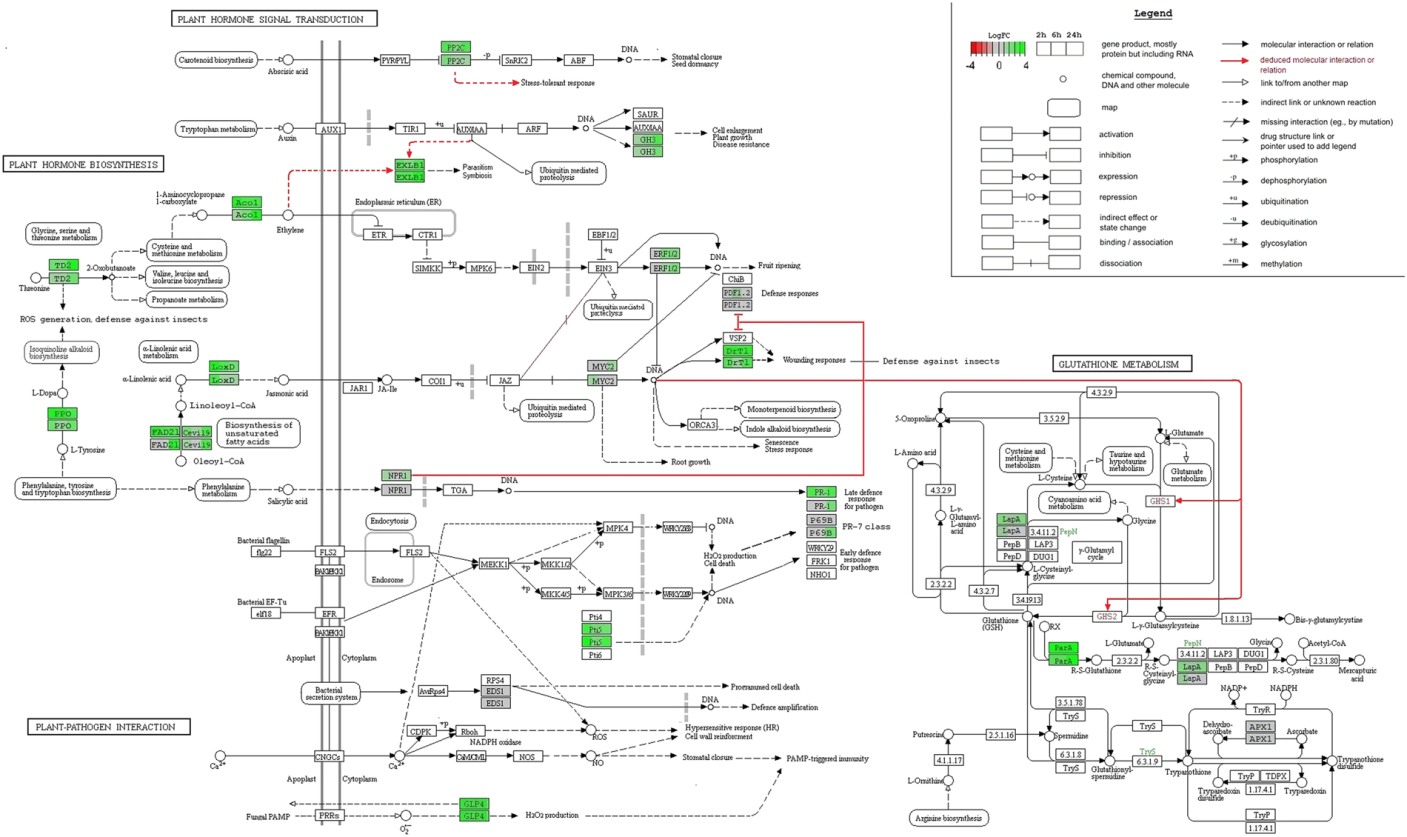
Pathway analysis of MBI600-tomato interactions. Log2 fold changes of gene expression level mapped onto several KEGG pathways^95–97^ by R package “Pathview”. Data included the qPCR estimates of all genes that were analyzed at 15 days-old tomato seedlings tissue (see Fig. 1 and Fig. 2). Target genes are described as boxes that have been divided into three sections and colored according to time points 2, 6 and 24 hpa and Log2FC scale respectively. Each gene consists of two boxes, the upper one refers to flg22 treatment while the lower to MBI600 CFCF treatment. The full names of genes are listed in Supplementary Table S1.

To summarize and comprehend the results that were extracted from the expression analysis of the 24 genes described in Figs. 1 and 2, an overview of the various signaling pathways in which these genes are involved is demonstrated in Fig. 5. In addition to the hormonal network, it was revealed that MBI600 elicitors also activated genes that are directly or indirectly implicated in abiotic stress responses and pest tolerance. Metabolic changes that led to such events could be regulated by glutathione metabolism and redox signaling.

Concerning the hormonal network, MBI600 elicitors suppressed ABA and enhanced auxin signaling pathways. The suppression of ABA-mediated signaling was suggested by the activation of *pp2c*, a negative regulator of ABA^28,29^ that has been associated with cold and salt tolerance^30–32^. Auxin-mediated responses are implied by the activation of *gh3*.8 and *exlb1*. Timing of upregulation (24 hpa) was in accordance with the role of *gh3*.8 as a late auxin-responsive gene^33^ which regulates plant development^34^. This gene has been associated with enhanced disease resistance in rice through the suppression of auxin signaling and expression of expansins, in a SA and JA independent manner^35^. EXLB1, an expansin-like protein, is a member of a family of small cell wall proteins^36^ that have the ability to non-enzymatically trigger a pH-dependent relaxation of the cell wall thus enabling cell expansion^37^. Expansins affect water loss and thus drought, salinity, and heat tolerance^38–40^. The elevated abundance of *exlb1* transcripts, despite *gh3*.*8* upregulation, could be explained by the fact that expansins are not solely regulated by auxin but also ethylene^37^.

In fact, components of Jasmonic acid (JA) and ethylene (ET) biosynthesis and signaling pathways were activated by MBI600 elicitors. Elevated transcript of *aco1*, an ACC oxidase (ACO) that participates in the final step of ET biosynthesis^41^, indicated the activation of the ET signaling pathway. ET is thought to signal ISR, in synergy with JA during root colonization by PGPR^6,42,43^. Such signaling involves regulation of the ethylene-responsive factor 1 (*erf1*) which is rapidly activated by ET or JA but requires both signaling pathways^44^ and acts as a transcription factor for the regulation of genes responsive to biotic or abiotic stress^45^. ET and JA synergy was confirmed by the increased expression levels of *erf1* following MBI600 CFCF or flg22 application. Another ET responsive factor that was activated early after CFCF treatment was *pti5* which is implicated in resistance gene-mediated recognition of bacterial pathogens^46^, independently of SA, ET and JA^47^ and is thought to activate SA-induced PR genes but not ET-regulated genes^48^.

The fact that *erf1* is negatively regulated by ET and positively regulated by JA^49^ suggests that MBI600 CFCF balanced the ET effect by producing JA. Indeed, MBI600 CFCF treatment led to the up-regulation of genes involved in JA biosynthesis such as *loxD*, a *fad*2-like (FAD2-L) and *cevi19*, a homolog of the *Arabidopsis* gene *fad2* lacking the putative third transmembrane domain^50^. Tomato lipoxygenase D (*loxD*) constitutes one of the first identified marker genes of the JA pathway and mediates defense against fungal and bacterial pathogens^51,52^ as well as herbivore attack^53^. Upstream of *loxD*, at the oxylipins pathway, function the fatty acid desaturases (*fad2* and *cevi19*). Unsaturated fatty acids have been thought to participate in plant responses to biotic and abiotic stress^54,55^. MBI600 CFCF was found to induce late up-regulation (at 24 hpa) of *fad2l* and *cevi19* in tomato seedlings. Unsaturated fatty acid accumulation in plants has been associated with *Pseudomonas putida*-mediated resistance to *Botrytis cinerea*^56^ and is associated with *Bacillus* sp. for the first time in this work. Activation of the JA signaling pathway was suggested by the expression of *myc2* which was mildly activated, early after CFCF treatment. MYC2 is a bHLH (basic helix-loop-helix) transcription factor that regulates JA-dependent gene expression^57^ such as *loxD*, *td2*, *lapA*, *parA*, *drt1*, and *pr1b1*, all of which was activated following CFCF application. Leucine aminopeptidase A (*lapA*), is a positive regulator of late wound responses^58^ and its up-regulation has been associated with water deficit, salinity and herbivore defense^59,60^. Threonine Deaminase (TD) functions in the chloroplast to catalyze the committed step in the biosynthesis of isoleucine and is induced by the JA signaling pathway in response to wounding and herbivory^61–63^. The proteinase inhibitor gene *drTI* is a small protein that belongs to the Kunitz-type PIs^64^. Generally, it is present in high concentrations in storage tissues but its expression can be detectable in leaves in response to an attack by insects or pathogens^65^. A similar response following PGPR challenge is reported for the first time.

It was recently found that MYC2 binds to promoters of the glutathione biosynthetic enzymes in order to activate their transcription during JA-mediated reoxygenation^66,67^. In support, MBI600 CFCF induced genes involved at the glutathione metabolism, such as *parA* and *lapA*. *parA* encodes for a glutathione *S*-transferase that catalyzes the conjugation of tripeptide (γ-Glu-Cys-Gly) glutathione (GSH) to a variety of substrates such as endobiotic and xenobiotic compounds for the detoxification and its expression is induced in response to biotic stress^68^. The fact that *lapA* also participates at the same biosynthetic pathway, that of mercapturic acid, indicates an activation of xenobiotic detoxification following CFCF treatment. Previous reports on the role of MYC2 as a master regulator of JA signaling^69^ are confirmed in this study.

In contrast to leaf application, seedling drench with MBI600 did not affect SA-responsive genes targeted in this study. Non-expressor of PR genes 1 (*npr1*) is activated by SA and has a dual function. Inside the nucleus, it acts as a transcriptional co-activator of a large set of defense effectors, including the PR proteins. It also facilitates cross-talk between SA and JA/ET signaling pathways in the cytosol, by suppressing the transcription of JA-responsive gene^70,71^. In accordance with previous findings^72^, flg22-mediated activation of *npr1* led to elevated transcript levels of *pr1b1*, a basic member of Pathogenesis Related protein PR-1. On the contrary, transcript levels of *npr1* were not modified following MBI600 CFCF treatment. Moreover, expression of *eds1*, which mediates SA biosynthesis and downstream signaling of SA responsive genes^73^ was not affected by CFCF treatment. This suggests that activation of *pr1b1* by CFCF is SA-independent and could be ET-responsive. *Pdf1*.*2* is a plant defensin involved in plant innate immune responses^74,75^. Its expression is regulated by the ERF1 transcription factor which acts downstream of the intersection between ET and JA pathways^76,77^. MBI600 CFCF had no effect on *pdf1*.*2* transcript levels while a mild activation occurred after flg22 treatment. This might be a consequence of the JA/ET synergistic action in which MYC2 repressed the expression of *pdf1*.*2*^14^. In support, flg22 treatment resulted in the up-regulation of *npr1* at 2 and 24 hpa but not at 6 hpa. This corresponded to an up-regulation of JA-responsive *pdf1*.*2* at 6 hpa but not at 2 and 24 hpa, thus confirming the cytosolic function of NPR1.

MBI600 CFCF was also found to activate, possible through the plant stress hormone network, oxidative burst components. Genes *ppo* and *glp4* that are, directly or indirectly, involved in redox signaling were up-regulated following CFCF treatment. Expression patterns were similar to those that occurred following flg22 treatment. PPOs catalyze the production of quinones which might have an antibacterial role^78^. Germin and germin-like proteins (GLPs) have mainly superoxide dismutase activity^79,80^ and function in basal host resistance^81^. Interestingly, transcript levels of ascorbate peroxidase 1 (*apx1*), which participates at the antioxidative response that reduces H_2_O_2_^82^, were not affected by CFCF (or flg22). Expression of *p69b* at 24 hpa, could thus be attributed to H_2_O_2_ accumulation. P69B is one of 15 subtilisin-like proteases (subtilases) of tomato^83,84^ and has been previously reported to be induced upon pathogen attack^85^. Subtilisin was up-regulated late following CFCF (but not flg22) treatment and it coincided rather than preceded pr1 expression, as previously reported^86^.

This is the first study to report the ability of a commercial BCA formulation (Serifel) to induce plant defense mechanisms and to time the signaling events that occurred following drench or spray application of tomato plants. MBI600 elicitors were equally capable of inducing defense mechanisms, suggesting their perception root and leaf receptors. Induction of systemic resistance upon leaf-BCA interaction suggests that certain PGPRs also activate defense in the phyllosphere. This has been overlooked as research has been focused on aseptic PGPR-root systems. A secondary, indirect mode of action gives additional value to successful bio-fungicides of the rapidly growing biopesticide market.

## Methods

### Plant growth conditions

Tomato (*Solanum lycopersicum*) seeds were pre-germinated on moist potting soil under greenhouse conditions (25 °C and 16 h photoperiod). After 10 days, plantlets were transplanted into 9 cm square pots filled with a three-element (N-K-P) fertilizer compost and were left to grow for two more weeks or until the developmental stage of four real leaves before starting with experiments.

### Application of formulations and MBI600 CFCF

Serifel is a WP formulation containing 9.9% of MBI600 endospores as the only active ingredient; no MBI600 metabolites are included and the base material has no direct or indirect antimicrobial effect. Serifel was applied either by irrigation (drench) or spraying (foliar) twice with a 7-day interval. Dosage was adjusted based on label claims; drench suspension was adjusted to 30 mg/L water while spray suspension at 30 mg (low dosage) or 1.25 g (suggested dosage) of WP in 1 L water. A water treatment was used as a control. Each treatment plot included 12 plants and experiments were repeated three times. Sampling was performed at different time points (0, 1, 2, 3, 5, 7, 8 and 10 dpa) and every sample was a pool of 4 leaves of 4 different plants (3 samples per treatment). Samples were frozen in liquid nitrogen and stored at −80 °C. CFCF was extracted from an overnight culture of Bam 600 (OD600 1.2) after centrifugation at 3000 rpm for 15 min at 23 °C by filtration through 0.45 μm bacteriostatic filters (Sarstedt). CFCF or water (control) were applied in tomato plants by spraying. Sampling was performed at four-time points (0, 1, 4 and 7 dpa). Samples were a pool consisting of 4 leaves of 4 different plants (3 samples per treatment). Samples were frozen in liquid nitrogen and stored at −80 °C.

### Application of MBI600 CFCF in tomato seedlings

A modified version of Frei Dit Frey *et al*. protocol was used^87^. In brief, fifteen days after germination on agar plates containing 1 × MS medium (Duchefa), under controlled conditions, tomato seedlings were transferred to liquid MS (2 seedlings/2 mL of medium per well in 24-well-plates). Three hours after transfer, the medium was supplied with flg22 peptide (Eurogentec) to a final concentration of 1 μΜ or with MBI600 CFCF to 1/5 v/v volume. Control treatment consisted of a 1/5 v/v LB + MS solution. Plantlets were collected at different time points after treatment (2, 6 and 24 h), frozen in liquid nitrogen and stored at −80 °C. Three independent experiments were performed. Each experiment included 2 biological replicates per treatment and each replicate was a pool of two seedlings, yielding a total number of tested 108 seedlings.

### Gene expression and data analysis

Total RNA was extracted from each sample using TRI reagent (Ambion) according to the manufacturer’s instructions. All subsequent steps for transcriptional level analysis and RT-qPCR were performed as described before^88^. Transcript levels of the target genes were quantified relatively to ubiquitin^89^. Primer sequences are listed in Supplementary Table S1.

qPCR analysis was performed using the method described in Steibel *et al*.^90^. Ct values of each factor combination were fitted to LMMs as dependent variables while treatments, genes and their interactions comprised the fixed factors. Samples nested within treatment and experiment were the random effects of the models. Log fold change of any specific gene for every treatment relative to the control treatment and its confidence intervals were calculated by formulating the contrasts of the model estimates according to equation 5 of the report of Steibel *et al*.^90^. Estimates of the Ct values of the *Ubi3* gene were used as reference. In cases where multiple plates were needed for the coverage of all samples, multi-run calibration was implemented with the use of replicated template samples across respective runs^91^. After normalization, calibration samples were excluded from the analysis. R programming language (https://www.R-project.org/) was implemented for the whole analysis.

### Microarray and data analysis

Microarray experiments followed the Minimum Information about a Microarray Experiment guidelines for international standardization and quality control of microarray experiments^92^. Two independent biological replicates were implemented for each time point of the analysis (6 and 24 h). For each replicate, a pool of two tomato seedlings was used as a source for total RNA extraction using TRI reagent (Ambion, Inc.) according to the manufacturer’s instructions. Target preparation and Hybridization was performed as described in Katsarou *et al*.^93^ on a 4 × 44 K tomato array (Agilent Technologies G2519F-022270). Further processing and statistical analysis were performed in R language programming environment with the implementation of the *limma* package^94^, principally as single channel. Background correction was done using the *background Correct* function, specifying for *normexp* method at offset 50, while normalization between arrays was performed by *quantile* method. Differential expression was assessed by an empirical Bayes approach with cut-offs for the Benjamini–Hochberg FDR-corrected P-values or log2(FC) values at 0.1 or 2 respectively. Alternatively, in order to cross-check the number and identity of selected features, hybridization data were also processed by a dual-channel approach with *Aquantile* method implemented for normalization between arrays. Gene expression data have been uploaded to the Gene Expression Omnibus with accession number GSE123589.

## Supplementary information

Supplementary Infromation
