# Supplementary material for: Bacillus amyloliquefaciens MBI600 differentially induces tomato defense signaling pathways depending on plant part and dose of application
Source: Sci Rep. 2019 Dec 13;9:19120. doi: 10.1038/s41598-019-55645-2 (PMC6910970; doi:10.1038/s41598-019-55645-2)
Supplement: Supplementary file 1 — Supplementary Infromation [file 41598_2019_55645_MOESM1_ESM.pdf]

Bacillus amyloliquefaciens MBI600 differentially induces tomato defense signaling pathways depending on plant part and dose of application

Anastasia Dimopoulou[1,2], Ioannis Theologidis[2], Burghard Liebmann[3], Kriton Kalantidis[1,2], Nikon Vassilakos[2], Nicholas Skandalis[2,4,*]

[1] *University of Crete, Dept. of Biology, 70013 Heraklion, Greece*
[2] *Institute of Molecular Biology and Biotechnology, FORTH, 100 N. Plastira str., 70013, Heraklion, Greece*
[3] Global R&D Biologicals, BASF SE, 67117 Limburgerhof, Germany
[4] *Keck School of Medicine of University of Southern California, HSC 1441 Eastlake Ave, Los Angeles 90033, CA, USA*

*Corresponding author: N. Skandalis; *skandali@usc.edu*

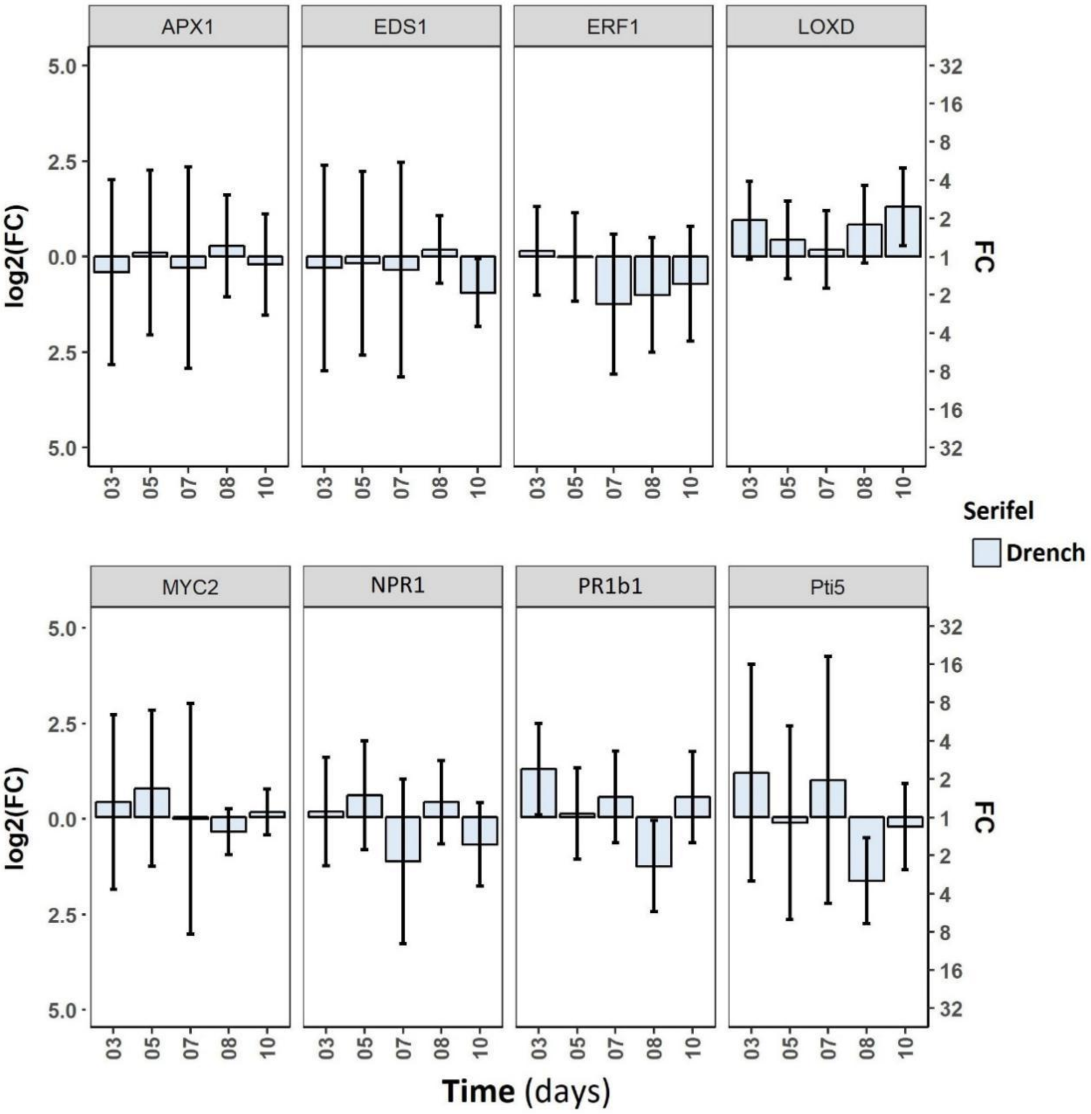

Supplementary Figure S1. Expression levels of defense-related marker genes of tomato plants in response to Serifel root application. 4 weeks-old plants were treated by drench with the suggested dosage of 0.03 g/L. of Serifel. Total RNA was extracted from leaf samples collected at 3, 5, 7, 8 and 10 dpa and used to perform RT-qPCR analysis using specific primers for eight defense genes Table S1.: 1. ***apx1***, ascorbate peroxidase 1; 2. ***eds1***, enhanced diseased susceptibility 1; 3. ***erf1***, ethylene response factor 1; 4. ***loxD***, lipoxygenase D; 5. ***myc2*** transcription factor; 6. ***npr1***, non-expressor of PR 1; 7. ***pr1b***, pathogenesis-related leaf protein 6 and 8. ***pti5***, Pto-interacting protein 5. Fold change (FC) represents the relative difference in expression between each treatment and control (water). *UBI3* was used as a reference gene. Bars indicate the 95% confidence interval (2*SE). Estimates whose confidence interval includes the baseline value 0 (1 in fold-change scale) are not significant from control at α=5%.

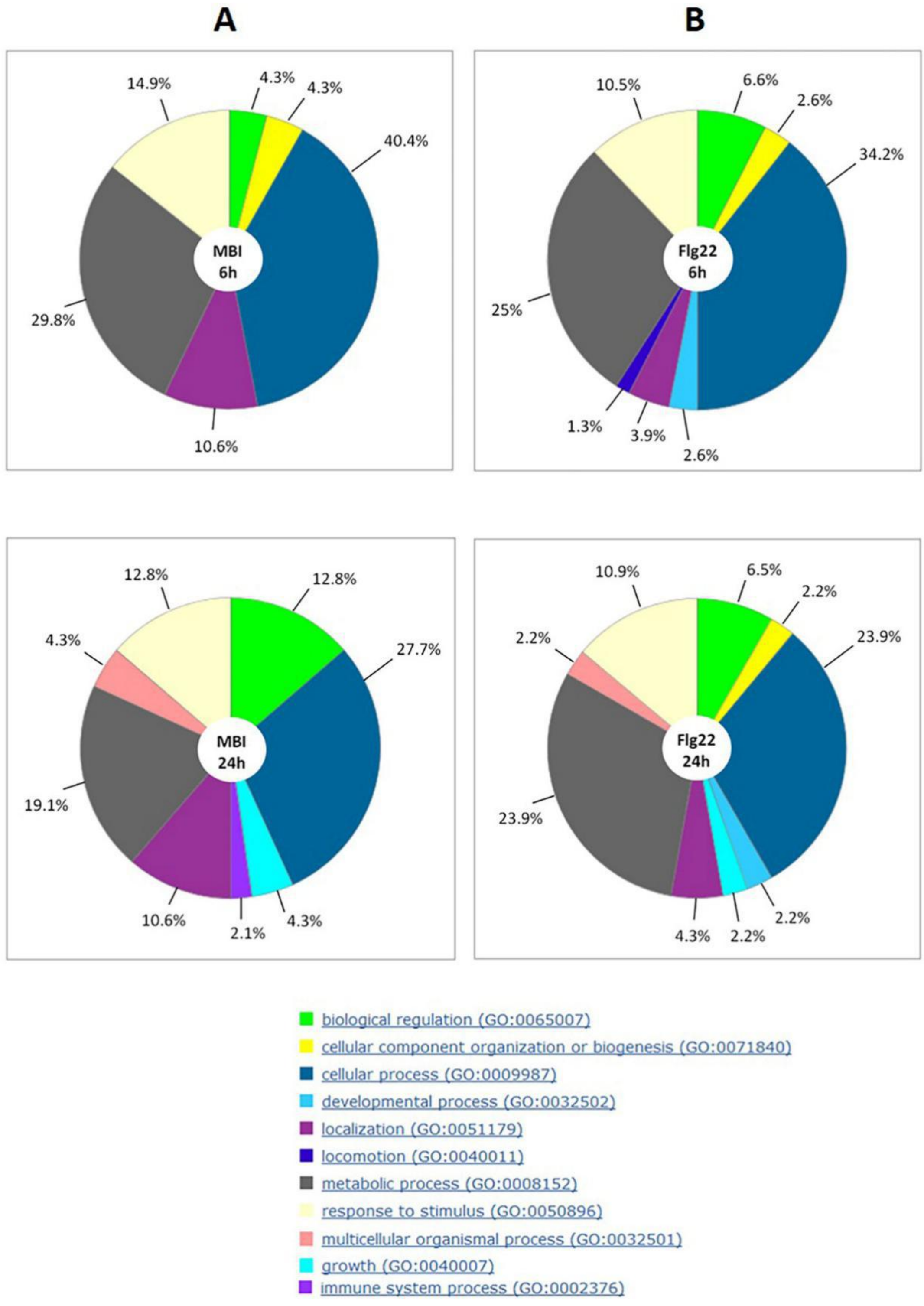

Supplementary Figure S2. Pie charts showing summarized Pantherdb analysis of the selected gene sets responding to either MBI600 CFCF or flg22 treatment. **A.** Biological process GO terms for *B. amyloliquefaciens* MBI600 CFCF at 6 and 24 hpa; **B.** Biological process GO term for flg22 at 6 and 24 hpa.

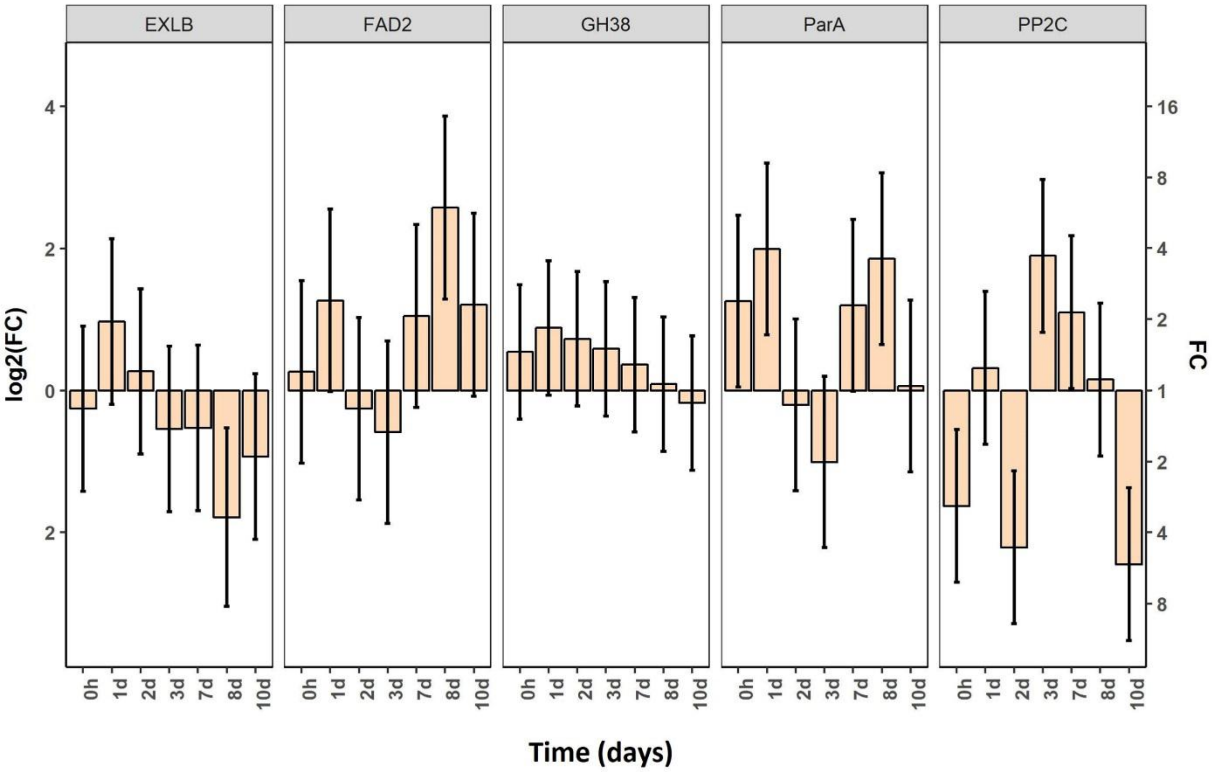

Supplementary Figure S3. Expression levels of novel genes in response to suggested dosage Serifel foliar application. Total RNA was extracted from leaf samples collected at 0, 1, 2, 3, 7, 8 and 10 dpa and used to perform RT-qPCR analysis using specific primers for the following genes Table S1.: 1. ***exlb1***, expansin-like B1; 2. ***fad2l***, delta12.-fatty-acid desaturase-like; 3. ***gh3.8***, IAA-amido synthetase; 4. ***parA***, glutathione S-transferase and 5. ***pp2c***, protein phosphatase 2C. Fold change (FC) represents the relative difference in expression between each treatment and control (water). *UBI3* was used as a reference gene. Bars indicate the 95% confidence interval (2*SE). Estimates whose confidence interval includes the baseline value 0 (1 in fold-change scale) are not significant from control at α=5%.

Name	SNG	Forward primer (5'-3')	Reverse primer (5'-3')
PR1b1	Solyc00g174340	GGTCGGGCACGTTGCA	GATCCAGTTGCCTACAGGACATA
ERF1	Solyc05g051200.1	TGGAGTTAGAAAGAGGCCATGG	CCCTCATTGATAATGCGGCTT
APX1	Solyc06g005160.3	ACTTCACGGAGCTTTTGAGTGG	CAGCATAGTCAGCAAAGAAGGC
MYC2	Solyc08g076930.1	TAGCCACACTGGAGGCAAGAT	CTAGGTCTAATTCCATGAGCGC
Pti5	Solyc02g077370.1	ATTCGCGATTCGGCTAGACAT	AGTAGTGCCTTAGCACCTCGCA
NIM1	Solyc07g040690.3	TGTGGGAAAGATAGCAGCAGC	GTCCACACAAACACACACATC
LoxD	Solyc03g122340.3	CCATCCTCACCACCCTCATC	TACTCGGGATCGTTCTCGTC
EDS1	Solyc06g071280.3	GATGCATTCAAGATTCAAAACACC	CAACATTTCAATGATCTCATCCCA
TD2	Solyc09g008670.2	GAGTGAAAAAGACGGGCTCAAA	CTATCAAACCCCCACCACCA
PPO	Solyc08g074680.2	GGTGACTAATGCTCCATGTCC	ATGTCCCATATTCTCCCCGTTT
ACO1	Solyc07g049530.2	CAAGATGACAAAGTGAGTGGC	ACATTCGTGTCCCGTCTGTTT
PIN2	Solyc03g020080.2	CATCTTCTGGATTGCCCA	ACACACAACTTGATGCCCAC
LAPA	Solyc00g187050.2<br>Solyc12g010040.1	TATTCTTGGACTGGGAACTGGA	GTGGACGCAATCTTTTTAGCC
EXLB1	Solyc08g077910.2	TGATGGCAAAGGAACACCTACT	CACCTTAACACCCTCGTTGCT
Cevi19	Solyc12g044950.1	TGGATTGCTCAAGGTTGTGTT	TGGTGACGACGATGACTATGTT
GLP4	Solyc01g102910.2	CTGAACCCACCTCATACTCAC	CCTACTGGGAAAACAAACACATC
DrTl	Solyc03g098720.2	AACATCGTCTGTCCCTTCCAG	GAAACCTTCCACCATCCACAC
NBS-LRR	Solyc07g056200.2	TATCCGCCTTATCAATACCCTGT	ATCTTGGATCTCCCTGTTCTTC
FAD2L	Solyc12g049030.1	AGAAAGCCATCCCTCCTCACT	GAACGATCCAATAAGCGAACC
P69B	Solyc08g079870.1	CCAACGCAACATTCTTCACTC	ATAATCATGCCAACACCTCCAG
GH3.8	Solyc01g107400.2	AGAGAAGTGGGATTACAGGAGAA	TTTACGGGACGGAGCAAATAC
PP2C	Solyc03g007230.2	TCGGGGTAGTAGGGATAACATCA	AAACAAGCAAGCAAGGCAGTAATG
ParA	Solyc07g056510.2	GTCCTAAACTTGTTGAATGGGC	GGAACCAAAACCTGTAAGCAGA
PDF1.2	XM_004242790 (NCBI)	AAAAAGTGGCAAGTGGAATGG	AATGGCAAGGTGAGTAGCAGTAA

Supplementary Table S1. List of primers used in this study

Figure 1-comparisons of all treatments against control (MS+LB)						
**Gene**	**Treatment**	**estimate (Log2FC)**	**SE**	**df**	**t.ratio**	**p.value**
**APX1**	flg22_02h	0.19	0.364697	181.16	0.521	0.603
	MBI600 CFCF_02h	0.4375	0.364697	181.16	1.200	0.2319
	flg22_06h	0.4725	0.364697	181.16	1.296	0.1968
	MBI600 CFCF_06h	0.45	0.364697	181.16	1.234	0.2188
	flg22_24h	0.7625	0.364697	181.16	2.091	0.0379
	MBI600 CFCF_24h	0.50375	0.364697	181.16	1.381	0.1689
**EDS1**	flg22_02h	0.31112313	0.250121	539.92	1.244	0.2141
	MBI600 CFCF_02h	0.47700024	0.250121	539.92	1.907	0.057
	flg22_06h	0.25624108	0.250121	539.92	1.024	0.3061
	MBI600 CFCF_06h	-0.18236256	0.250121	539.92	-0.729	0.4663
	flg22_24h	0.52942634	0.250121	539.92	2.117	0.0347
	MBI600 CFCF_24h	0.38164044	0.250121	539.92	1.526	0.1276
**ERF1**	flg22_02h	0.79728102	0.345529	259.53	2.307	0.0218
	MBI600 CFCF_02h	0.23613811	0.345529	259.53	0.683	0.495
	flg22_06h	2.22802186	0.345529	259.53	6.448	<.0001
	MBI600 CFCF_06h	0.86789656	0.345529	259.53	2.512	0.0126
	flg22_24h	2.29856729	0.345529	259.53	6.652	<.0001
	MBI600 CFCF_24h	1.68649030	0.345529	259.53	4.881	<.0001
**LOXD**	flg22_02h	3.76796436	0.449788	370.97	8.377	<.0001
	MBI600 CFCF_02h	2.46222973	0.502878	579.64	4.896	<.0001
	flg22_06h	2.50995636	0.449788	370.97	5.580	<.0001
	MBI600 CFCF_06h	0.88601653	0.449788	370.97	1.970	0.0496
	flg22_24h	1.92176278	0.449788	370.97	4.273	<.0001
	MBI600 CFCF_24h	1.08266671	0.449788	370.97	2.407	0.0166
**MYC2**	flg22_02h	0.73113251	0.238662	599.42	3.063	0.0023
	**MBI600 CFCF_02h**	**0.93018389**	**0.238662**	**599.42**	**3.897**	**0.0001**
	flg22_06h	0.71407842	0.238662	599.42	2.992	0.0029
	MBI600 CFCF_06h	0.10198545	0.238662	599.42	0.427	0.6693
	flg22_24h	0.97425604	0.238662	599.42	4.082	0.0001
	MBI600 CFCF_24h	0.06257629	0.238662	599.42	0.262	0.7933
**NPR1**	flg22_02h	1.07950973	0.411551	164.41	2.623	0.0095
	MBI600 CFCF_02h	0.68082595	0.411551	164.41	1.654	0.1
	flg22_06h	0.21170878	0.411551	164.41	0.514	0.6077
	MBI600 CFCF_06h	-0.63419533	0.411551	164.41	-1.541	0.1252
	flg22_24h	0.95125914	0.411551	164.41	2.311	0.0221
	MBI600 CFCF_24h	0.34426546	0.411551	164.41	0.837	0.4041
**PR1b1**	flg22_02h	3.05919719	0.760181	28.69	4.024	0.0004
	MBI600 CFCF_02h	0.07119012	0.760181	28.69	0.094	0.926
	flg22_06h	1.76006413	0.760181	28.69	2.315	0.028
	MBI600 CFCF_06h	-0.29770064	0.760181	28.69	-0.392	0.6982
	flg22_24h	2.72692179	0.760181	28.69	3.587	0.0012

	MBI600 CFCF_24h	2.05903244	0.760181	28.69	2.709	0.0113
**Pti5**	flg22_02h	3.00500000	0.496224	99	6.056	<.0001
	MBI600 CFCF_02h	3.57625000	0.496224	99	7.207	<.0001
	flg22_06h	2.00250000	0.496224	99	4.035	0.0001
	MBI600 CFCF_06h	2.01750000	0.496224	99	4.066	0.0001
	flg22_24h	2.19750000	0.496224	99	4.428	<.0001
	MBI600 CFCF_24h	2.98000000	0.496224	99	6.005	<.0001
**Figure 2-comparisons of all treatments against control (MS+LB)**						
**Gene**	**Treatment**	**estimate (Log2FC)**	**SE**	**df**	**t.ratio**	**p.value**
**ACO1**	flg22_02h	4.067500	0.404014	99	10.068	<.0001
	MBI600 CFCF_02h	1.910000	0.404014	99	4.728	<.0001
	flg22_06h	3.036250	0.404014	99	7.515	<.0001
	MBI600 CFCF_06h	2.073750	0.404014	99	5.133	<.0001
	**flg22_24h**	**2.671250**	**0.404014**	**99**	**6.612**	**<.0001**
	**MBI600 CFCF_24h**	**4.020000**	**0.404014**	**99**	**9.950**	**<.0001**
**Cevi19**	flg22_02h	2.173750	0.424465	99	5.121	<.0001
	**MBI600 CFCF_02h**	**1.616250**	**0.424465**	**99**	**3.808**	**0.0002**
	flg22_06h	1.277500	0.424465	99	3.010	0.0033
	MBI600 CFCF_06h	0.32625	0.424465	99	0.769	0.444
	flg22_24h	2.532500	0.424465	99	5.966	<.0001
	**MBI600 CFCF_24h**	**2.150000**	**0.424465**	**99**	**5.065**	**<.0001**
**DrTi**	flg22_02h	5.715000	0.543315	99	10.519	<.0001
	**MBI600 CFCF_02h**	**3.058750**	**0.543315**	**99**	**5.630**	**<.0001**
	flg22_06h	4.665000	0.543315	99	8.586	<.0001
	MBI600 CFCF_06h	1.170000	0.543315	99	2.153	0.0337
	flg22_24h	8.663750	0.543315	99	15.946	<.0001
	**MBI600 CFCF_24h**	**3.077500**	**0.543315**	**99**	**5.664**	**<.0001**
**EXLB1**	flg22_02h	2.601250	0.383119	99	6.790	<.0001
	MBI600 CFCF_02h	3.067500	0.383119	99	8.007	<.0001
	flg22_06h	1.835000	0.383119	99	4.790	<.0001
	MBI600 CFCF_06h	3.228750	0.383119	99	8.428	<.0001
	flg22_24h	2.773750	0.383119	99	7.240	<.0001
	**MBI600 CFCF_24h**	**4.166250**	**0.383119**	**99**	**10.875**	**<.0001**
**FAD2**	flg22_02h	3.183750	0.788768	99	4.036	0.0001
	MBI600 CFCF_02h	1.028750	0.788768	99	1.304	0.1952
	flg22_06h	3.711250	0.788768	99	4.705	<.0001
	MBI600 CFCF_06h	0.6525	0.788768	99	0.827	0.4101
	flg22_24h	2.933750	0.788768	99	3.719	0.0003
	**MBI600 CFCF_24h**	**4.847500**	**0.788768**	**99**	**6.146**	**<.0001**
**GH3.8**	flg22_02h	1.130000	0.215865	99	5.235	<.0001
	MBI600 CFCF_02h	0.95625	0.215865	99	4.430	<.0001
	flg22_06h	1.850000	0.215865	99	8.570	<.0001
	MBI600 CFCF_06h	1.501250	0.215865	99	6.955	<.0001

	flg22_24h	3.051250	0.215865	99	14.135	<.0001
	**MBI600 CFCF_24h**	**2.112500**	**0.215865**	**99**	**9.786**	**<.0001**
**GLP4**	flg22_02h	1.698750	0.342559	97.8	4.959	<.0001
	**MBI600 CFCF_02h**	**2.635000**	**0.342559**	**97.8**	**7.692**	**<.0001**
	flg22_06h	1.802880	0.350027	98.05	5.151	<.0001
	MBI600 CFCF_06h	2.358750	0.342559	97.8	6.886	<.0001
	flg22_24h	1.707500	0.342559	97.8	4.985	<.0001
	MBI600 CFCF_24h	1.366250	0.342559	97.8	3.988	0.0001
**LapA**	flg22_02h	1.972500	0.418009	99	4.719	<.0001
	**MBI600 CFCF_02h**	**1.341250**	**0.418009**	**99**	**3.209**	**0.0018**
	flg22_06h	1.543750	0.418009	99	3.693	0.0004
	MBI600 CFCF_06h	0.645	0.418009	99	1.543	0.126
	flg22_24h	1.942500	0.418009	99	4.647	<.0001
	MBI600 CFCF_24h	-0.37375	0.418009	99	-0.894	0.3734
**NBSLRR**	flg22_02h	0.885	0.33543	99	2.638	0.0097
	**MBI600 CFCF_02h**	**0.7425**	**0.33543**	**99**	**2.214**	**0.0292**
	flg22_06h	1.477500	0.33543	99	4.405	<.0001
	MBI600 CFCF_06h	-0.3425	0.33543	99	-1.021	0.3097
	flg22_24h	1.726250	0.33543	99	5.146	<.0001
	**MBI600 CFCF_24h**	**1.260000**	**0.33543**	**99**	**3.756**	**0.0003**
**P69b**	flg22_02h	0.7925	0.712864	99	1.112	0.269
	MBI600 CFCF_02h	-0.32125	0.712864	99	-0.451	0.6532
	flg22_06h	0.61875	0.712864	99	0.868	0.3875
	MBI600 CFCF_06h	-0.875	0.712864	99	-1.227	0.2226
	flg22_24h	0.76125	0.712864	99	1.068	0.2882
	**MBI600 CFCF_24h**	**2.045000**	**0.712864**	**99**	**2.869**	**0.005**
**ParA**	flg22_02h	3.931250	0.363329	98.04	10.820	<.0001
	MBI600 CFCF_02h	1.977975	0.371269	98.27	5.328	<.0001
	flg22_06h	3.182500	0.363329	98.04	8.759	<.0001
	MBI600 CFCF_06h	2.247500	0.363329	98.04	6.186	<.0001
	**flg22_24h**	**2.648750**	**0.363329**	**98.04**	**7.290**	**<.0001**
	**MBI600 CFCF_24h**	**5.500000**	**0.363329**	**98.04**	**15.138**	**<.0001**
**PIN2**	flg22_02h	5.051250	0.621562	98.09	8.127	<.0001
	**MBI600 CFCF_02h**	**3.278334**	**0.634788**	**98.53**	**5.164**	**<.0001**
	flg22_06h	4.576250	0.621562	98.09	7.362	<.0001
	MBI600 CFCF_06h	2.107500	0.621562	98.09	3.391	0.001
	flg22_24h	8.783750	0.621562	98.09	14.132	<.0001
	**MBI600 CFCF_24h**	**3.850000**	**0.621562**	**98.09**	**6.194**	**<.0001**
**PP2C**	flg22_02h	2.421250	0.331072	99	7.313	<.0001
	**MBI600 CFCF_02h**	**1.952500**	**0.331072**	**99**	**5.898**	**<.0001**
	flg22_06h	1.730000	0.331072	99	5.225	<.0001
	MBI600 CFCF_06h	1.572500	0.331072	99	4.750	<.0001
	flg22_24h	2.993750	0.331072	99	9.043	<.0001
	MBI600 CFCF_24h	0.9575	0.331072	99	2.892	0.0047

**PPO**	flg22_02h	3.946250	0.430586	98.04	9.165	<.0001
	**MBI600 CFCF_02h**	**2.017500**	**0.430586**	**98.04**	**4.685**	**<.0001**
	flg22_06h	3.556250	0.430586	98.04	8.259	<.0001
	MBI600 CFCF_06h	1.175145	0.439928	98.33	2.671	0.0088
	flg22_24h	2.470000	0.430586	98.04	5.736	<.0001
	MBI600 CFCF_24h	2.032500	0.430586	98.04	4.720	<.0001
**TD2**	flg22_02h	2.720000	0.627535	99	4.334	<.0001
	MBI600 CFCF_02h	1.518750	0.627535	99	2.420	0.0173
	flg22_06h	2.707500	0.627535	99	4.314	<.0001
	MBI600 CFCF_06h	1.551250	0.627535	99	2.472	0.0151
	flg22_24h	8.711250	0.627535	99	13.882	<.0001
	**MBI600 CFCF_24h**	**2.327500**	**0.627535**	**99**	**3.709**	**0.0003**
**PDF1.2**	flg22_02h	0.1275842	0.383772	99	0.332	0.7403
	MBI600 CFCF_02h	0.3764872	0.383772	99	0.981	0.329
	flg22_06h	0.8822066	0.383772	99	2.299	0.0236
	MBI600 CFCF_06h	0.1896351	0.383772	99	0.494	0.6223
	flg22_24h	-0.1882538	0.383772	99	-0.491	0.6248
	MBI600 CFCF_24h	-0.177165	0.383772	99	-0.462	0.6454
**Figure 3-comparisons of all treatments against control (water)**						
**Gene**	**Treatment**	**estimate (Log2FC)**	**SE**	**df**	**t.ratio**	**p.value**
**APX1**	MBI600 CFCF_00h	0.0775	0.142543	1332.7	0.544	0.5867
	MBI600 CFCF_01d	-0.0725	0.142543	1332.7	-0.509	0.6111
	MBI600 CFCF_04d	0.0775	0.142543	1040.2	0.544	0.5868
	MBI600 CFCF_07d	0.398333333	0.153964	1332.7	2.587	0.0098
**EDS1**	MBI600 CFCF_00h	-0.33125	0.301388	262.78	-1.099	0.2727
	**MBI600 CFCF_01d**	**1.187500000**	**0.301388**	**262.78**	**3.940**	**0.0001**
	MBI600 CFCF_04d	0.255	0.301388	206.19	0.846	0.3985
	**MBI600 CFCF_07d**	**-0.865416667**	**0.325536**	**262.78**	**-2.658**	**0.0083**
**ERF1**	**MBI600 CFCF_00h**	**0.92**	**0.303752**	**272.86**	**3.029**	**0.0027**
	MBI600 CFCF_01d	-0.2725	0.303752	272.86	-0.897	0.3704
	MBI600 CFCF_04d	1.207500000	0.303752	214.75	3.975	0.0001
	MBI600 CFCF_07d	0.25875	0.32809	272.86	0.789	0.431
**LOXD**	MBI600 CFCF_00h	0.24	0.362355	171.03	0.662	0.5086
	**MBI600 CFCF_01d**	**0.74125**	**0.362355**	**171.03**	**2.046**	**0.0423**
	MBI600 CFCF_04d	-1.262500000	0.362355	134.57	-3.484	0.0007
	**MBI600 CFCF_07d**	**-1.253333333**	**0.391388**	**171.03**	**-3.202**	**0.0016**
**MYC2**	**MBI600 CFCF_00h**	**0.72**	**0.319923**	**237.31**	**2.251**	**0.0253**
	MBI600 CFCF_01d	0.32375	0.319923	237.31	1.012	0.3126
	MBI600 CFCF_04d	-0.40875	0.319923	186.66	-1.278	0.203
	**MBI600 CFCF_07d**	**-1.170416667**	**0.345557**	**237.31**	**-3.387**	**0.0008**
**NPR1**	MBI600 CFCF_00h	-0.071742298	0.455641	82.97	-0.157	0.8753
	MBI600 CFCF_01d	0.869582653	0.455641	82.97	1.908	0.0598
	MBI600 CFCF_04d	0.560828532	0.484251	83.4	1.158	0.2501

	**MBI600 CFCF_07d**	**-1.065240699**	**0.492149**	**82.97**	**-2.164**	**0.0333**
**PR1b1**	MBI600 CFCF_00h	0.827831163	0.498389	73.16	1.661	0.101
	MBI600 CFCF_01d	0.007516386	0.487869	67.17	0.015	0.9878
	MBI600 CFCF_04d	1.137472869	0.487869	52.99	2.332	0.0236
	MBI600 CFCF_07d	0.322391717	0.526959	67.17	0.612	0.5427
**Pti5**	**MBI600 CFCF_00h**	**1.091492175**	**0.477663**	**82.05**	**2.285**	**0.0249**
	MBI600 CFCF_01d	-0.670784473	0.477663	82.05	-1.404	0.164
	MBI600 CFCF_04d	-0.233710074	0.50725	82.57	-0.461	0.6462
	MBI600 CFCF_07d	-0.162029821	0.515935	82.05	-0.314	0.7543
**Figure 4A-comparisons of all treatments against control (water)**						
**Gene**	**Treatment**	**estimate (Log2FC)**	**SE**	**df**	**t.ratio**	**p.value**
**APX1**	Serifel_1st.spray_00h	-0.15666667	13.181	20	-0.119	0.9066
	Serifel_1st.spray_07d	0.03666667	13.181	20	0.028	0.9781
	Serifel_1st.spray_03d	-1.6433333	1.526.459	16.14	-1.077	0.2975
	Serifel_1st.spray_05d	0.6333333	1.079.369	16.14	0.587	0.5655
	Serifel_1st.spray_08d	-0.19166667	0.664071	75	-0.289	0.7737
	Serifel_1st.spray_10d	-1.23500000	0.664071	75	-1.860	0.0668
	Serifel_2nd.spray_08d	0.13	0.664071	75	0.196	0.8453
	Serifel_2nd.spray_10d	0.01333333	0.664071	75	0.02	0.984
**EDS1**	Serifel_1st.spray_00h	-0.5533333	1.404.289	20	-0.394	0.6977
	Serifel_1st.spray_07d	0.3	1.404.289	20	0.214	0.833
	Serifel_1st.spray_03d	-1.7966667	1.699.503	16.17	-1.057	0.306
	Serifel_1st.spray_05d	0.8333333	1.201.730	16.17	0.693	0.4979
	Serifel_1st.spray_08d	-0.1416667	0.442983	75	-0.32	0.75
	Serifel_1st.spray_10d	-0.1816667	0.442983	75	-0.41	0.6829
	Serifel_2nd.spray_08d	-0.2866667	0.442983	75	-0.647	0.5195
	Serifel_2nd.spray_10d	-0.3983333	0.442983	75	-0.899	0.3714
**ERF1**	Serifel_1st.spray_00h	-0.09333333	0.917292	48	-0.102	0.9194
	Serifel_1st.spray_07d	-0.235	0.917292	48	-0.256	0.7989
	Serifel_1st.spray_03d	0.26	0.578209	58	0.45	0.6546
	Serifel_1st.spray_05d	0.6233333	0.578209	58	1.078	0.2855
	Serifel_1st.spray_08d	0.265	0.750829	75	0.353	0.7251
	Serifel_1st.spray_10d	-1.4483333	0.750829	75	-1.929	0.0575
	Serifel_2nd.spray_08d	0.4433333	0.750829	75	0.59	0.5567
	Serifel_2nd.spray_10d	-0.135	0.750829	75	-0.18	0.8578
**LOXD**	Serifel_1st.spray_00h	-0.73166667	0.58809	125	-1.244	0.2158
	Serifel_1st.spray_03d	-0.71166667	0.509301	125	-1.397	0.1648
	Serifel_1st.spray_05d	-0.01333333	0.509301	125	-0.026	0.9792
	Serifel_1st.spray_07d	-0.08666667	0.509301	125	-0.17	0.8652
	Serifel_1st.spray_08d	0.16916667	0.509301	125	0.332	0.7403
	Serifel_1st.spray_10d	-0.30416667	0.509301	125	-0.597	0.5514
	Serifel_1st.spray_08d	0.31083333	0.509301	125	0.61	0.5428
	Serifel_1st.spray_10d	-0.69416667	0.509301	125	-1.363	0.1753

**MYC2**	Serifel_1st.spray_00h	-0.63666667	1.511.188	18	-0.421	0.6785
	Serifel_1st.spray_07d	0.43333333	1.511.188	18	0.287	0.7776
	Serifel_1st.spray_03d	0.1966667	1.445.748	16.05	0.136	0.8935
	Serifel_1st.spray_05d	1.2500000	1.022.298	16.05	1.223	0.2391
	Serifel_1st.spray_08d	0.233333333	0.299917	74	0.778	0.4391
	Serifel_1st.spray_10d	0.283333333	0.299917	74	0.945	0.3479
	Serifel_2nd.spray_08d	0.55466093	0.307747	74.06	1.802	0.0756
	Serifel_2nd.spray_10d	0.008333333	0.299917	74	0.028	0.9779
**NPR1**	Serifel_1st.spray_00h	-0.1394385	1.105.980	47.01	-0.126	0.9002
	Serifel_1st.spray_07d	-0.25	1.078.301	47	-0.232	0.8177
	Serifel_1st.spray_03d	-0.745	0.712392	58	-1.046	0.3
	**Serifel_1st.spray_05d**	**1.8300000**	**0.712392**	**58**	**2.569**	**0.0128**
	Serifel_1st.spray_08d	0.6383333	0.544429	80	1.172	0.2445
	Serifel_1st.spray_10d	-0.37	0.544429	80	-0.68	0.4987
	Serifel_2nd.spray_08d	0.9666667	0.544429	80	1.776	0.0796
	Serifel_2nd.spray_10d	-0.6116667	0.544429	80	-1.124	0.2646
**PR1b1**	Serifel_1st.spray_00h	-12.183.333	0.690887	125	-1.763	0.0803
	Serifel_1st.spray_03d	0.8691667	0.598325	125	1.453	0.1488
	**Serifel_1st.spray_05d**	**2.3341667**	**0.598325**	**125**	**3.901**	**0.0002**
	Serifel_1st.spray_07d	2.5625000	0.598325	125	4.283	<.0001
	Serifel_1st.spray_08d	1.4250000	0.598325	125	2.382	0.0187
	Serifel_1st.spray_10d	0.23	0.598325	125	0.384	0.7013
	**Serifel_2nd.spray_08d**	**1.9566667**	**0.598325**	**125**	**3.270**	**0.0014**
	Serifel_2nd.spray_10d	-1.1166667	0.598325	125	-1.866	0.0643
**Pti5**	Serifel_1st.spray_00h	-1.5733333	1.617.983	16.98	-0.972	0.3445
	Serifel_1st.spray_07d	1.0148813	1.716.641	17.35	0.591	0.562
	Serifel_1st.spray_03d	-0.6666667	1.794.584	16.18	-0.371	0.7151
	Serifel_1st.spray_05d	-0.2733333	1.268.963	16.18	-0.215	0.8321
	Serifel_1st.spray_08d	-0.3666667	0.561781	75	-0.653	0.516
	Serifel_1st.spray_10d	0.2033333	0.561781	75	0.362	0.7184
	Serifel_2nd.spray_08d	-0.7683333	0.561781	75	-1.368	0.1755
	Serifel_2nd.spray_10d	-1.0083333	0.561781	75	-1.795	0.0767
**Figure 4B-comparisons of all treatments against control (water)**						
**Gene**	**Treatment**	**estimate (Log2FC)**	**SE**	**df**	**t.ratio**	**p.value**
**APX1**	Serifel_1st.spray_00h	-0.446666667	0.327357	446.56	-1.364	0.1731
	Serifel_1st.spray_01d	0.146666667	0.327357	446.56	0.448	0.6543
	Serifel_1st.spray_02d	-0.178333333	0.327357	446.56	-0.545	0.5862
	Serifel_1st.spray_03d	0.596666667	0.327357	446.56	1.823	0.069
	Serifel_1st.spray_07d	0.105833333	0.231477	446.56	0.457	0.6477
	Serifel_1st.spray_08d	1.150000000	0.327357	446.56	3.513	0.0005
	Serifel_1st.spray_10d	0.150891052	0.336849	413.76	0.448	0.6544
	Serifel_2nd.spray_07d	0.61	0.2835	446.56	2.152	0.032
	**Serifel_2nd.spray_08d**	**1.110000000**	**0.327357**	**446.56**	**3.391**	**0.0008**

	Serifel_2nd.spray_10d	-0.04	0.327357	446.56	-0.122	0.9028
EDS1	Serifel_1st.spray_00h	-0.506666667	0.329454	452.13	-1.538	0.1248
	**Serifel_1st.spray_01d**	**0.81**	**0.329454**	**452.13**	**2.459**	**0.0143**
	Serifel_1st.spray_02d	-0.355	0.329454	452.13	-1.078	0.2818
	Serifel_1st.spray_03d	0.125	0.329454	452.13	0.379	0.7046
	Serifel_1st.spray_07d	0.356666667	0.23296	452.13	1.531	0.1265
	Serifel_1st.spray_08d	0.516666667	0.329454	452.13	1.568	0.1175
	Serifel_1st.spray_10d	-0.05	0.329454	373.66	-0.152	0.8795
	Serifel_2nd.spray_07d	1.278333333	0.285316	452.13	4.480	<.0001
	Serifel_2nd.spray_08d	0.515	0.329454	452.13	1.563	0.1187
	**Serifel_2nd.spray_10d**	**-0.68**	**0.329454**	**452.13**	**-2.064**	**0.0396**
ERF1	Serifel_1st.spray_00h	0.178333333	0.372812	332.98	0.478	0.6327
	**Serifel_1st.spray_01d**	**1.763333333**	**0.372812**	**332.98**	**4.730**	**<.0001**
	Serifel_1st.spray_02d	-0.356666667	0.372812	332.98	-0.957	0.3394
	Serifel_1st.spray_03d	0.74	0.372812	332.98	1.985	0.048
	Serifel_1st.spray_07d	0.329166667	0.263618	332.98	1.249	0.2127
	**Serifel_1st.spray_08d**	**-0.263333333**	**0.372812**	**332.98**	**-0.706**	**0.4805**
	**Serifel_1st.spray_10d**	**-1.036666667**	**0.372812**	**275.19**	**-2.781**	**0.0058**
	Serifel_2nd.spray_07d	0.583333333	0.322865	332.98	1.807	0.0717
	**Serifel_2nd.spray_08d**	**-0.146666667**	**0.372812**	**332.98**	**-0.393**	**0.6943**
	**Serifel_2nd.spray_10d**	**-0.797004102**	**0.383275**	**371.97**	**-2.079**	**0.0383**
LOXD	**Serifel_1st.spray_00h**	**1.010000000**	**0.400134**	**270.41**	**2.524**	**0.0122**
	Serifel_1st.spray_01d	-0.173333333	0.400134	270.41	-0.433	0.6652
	Serifel_1st.spray_02d	-0.31	0.400134	270.41	-0.775	0.4392
	Serifel_1st.spray_03d	-0.25	0.400134	270.41	-0.625	0.5326
	**Serifel_1st.spray_07d**	**0.820833333**	**0.282938**	**270.41**	**2.901**	**0.004**
	**Serifel_1st.spray_08d**	**1.876666667**	**0.400134**	**270.41**	**4.690**	**<.0001**
	Serifel_1st.spray_10d	-0.391666667	0.400134	223.48	-0.979	0.3287
	Serifel_2nd.spray_07d	0.899166667	0.346526	270.41	2.595	0.01
	Serifel_2nd.spray_08d	1.973333333	0.400134	270.41	4.932	<.0001
	Serifel_2nd.spray_10d	-0.331666667	0.400134	270.41	-0.829	0.4079
MYC2	Serifel_1st.spray_00h	-0.265	0.237103	934	-1.118	0.264
	Serifel_1st.spray_01d	0.266666667	0.237103	934	1.125	0.261
	Serifel_1st.spray_02d	-0.491666667	0.237103	934	-2.074	0.0384
	Serifel_1st.spray_03d	0.085	0.237103	934	0.358	0.7201
	**Serifel_1st.spray_07d**	**0.6875**	**0.167657**	**934**	**4.101**	**<.0001**
	**Serifel_1st.spray_08d**	**0.343333333**	**0.237103**	**934**	**1.448**	**0.1479**
	Serifel_1st.spray_10d	-0.208333333	0.237103	771.9	-0.879	0.3799
	**Serifel_2nd.spray_07d**	**0.619166667**	**0.205337**	**934**	**3.015**	**0.0026**
	**Serifel_2nd.spray_08d**	**0.768333333**	**0.237103**	**934**	**3.241**	**0.0012**
	Serifel_2nd.spray_10d	-0.141666667	0.237103	934	-0.597	0.5503
NPR1	Serifel_1st.spray_00h	0.3	0.453834	195.38	0.661	0.5094
	**Serifel_1st.spray_01d**	**1.060000000**	**0.453834**	**195.38**	**2.336**	**0.0205**
	Serifel_1st.spray_02d	0.275	0.453834	195.38	0.606	0.5453

	**Serifel_1st.spray_03d**	**1.228333333**	**0.453834**	**195.38**	**2.707**	**0.0074**
	Serifel_1st.spray_07d	0.160490077	0.330076	218.68	0.486	0.6273
	Serifel_1st.spray_08d	0.083926974	0.478498	241.44	0.175	0.8609
	Serifel_1st.spray_10d	-0.038333333	0.453834	161.47	-0.084	0.9328
	Serifel_2nd.spray_07d	1.340490077	0.400551	210.77	3.347	0.001
	Serifel_2nd.spray_08d	0.121700743	0.466329	217.8	0.261	0.7944
	Serifel_2nd.spray_10d	-0.673333333	0.453834	195.38	-1.484	0.1395
**PR1b1**	Serifel_1st.spray_00h	-0.363333333	0.531344	101.36	-0.684	0.4957
	**Serifel_1st.spray_01d**	**2730000000**	**0.531344**	**101.36**	**5.138**	**<.0001**
	Serifel_1st.spray_02d	0.001666667	0.531344	101.36	0.003	0.9975
	Serifel_1st.spray_03d	0.121666667	0.531344	101.36	0.229	0.8193
	**Serifel_1st.spray_07d**	**2.708333333**	**0.375717**	**101.36**	**7.208**	**<.0001**
	**Serifel_1st.spray_08d**	**1.576666667**	**0.531344**	**101.36**	**2.967**	**0.0037**
	**Serifel_1st.spray_10d**	**1.310000000**	**0.531344**	**83.77**	**2.465**	**0.0157**
	Serifel_2nd.spray_07d	2.745000000	0.460157	101.36	5.965	<.0001
	Serifel_2nd.spray_08d	0.975	0.531344	101.36	1.835	0.0694
	Serifel_2nd.spray_10d	0.85320431	0.54669	113.59	1.561	0.1214
**Pti5**	Serifel_1st.spray_00h	0.183333333	0.484338	156.83	0.379	0.7056
	**Serifel_1st.spray_01d**	**1.211666667**	**0.484338**	**156.83**	**2.502**	**0.0134**
	Serifel_1st.spray_02d	0.446666667	0.484338	156.83	0.922	0.3578
	Serifel_1st.spray_03d	0.325	0.484338	156.83	0.671	0.5032
	**Serifel_1st.spray_07d**	**1.105000000**	**0.342478**	**156.83**	**3.226**	**0.0015**
	**Serifel_1st.spray_08d**	**2.821666667**	**0.484338**	**156.83**	**5.826**	**<.0001**
	Serifel_1st.spray_10d	0.538333333	0.507978	156.83	1.060	0.2909
	Serifel_2nd.spray_07d	2.164166667	0.419449	156.83	5.160	<.0001
	Serifel_2nd.spray_08d	2.766666667	0.484338	156.83	5.712	<.0001
	Serifel_2nd.spray_10d	-0.356666667	0.484338	156.83	-0.736	0.4626

Supplementary Figure S1-comparisons of all treatments against control (water)

Gene	Treatment	estimate (Log2FC)	SE	df	t.ratio	p.value
**APX1**	Serifel_drench_07d	-0.29666667	13.181	20	-0.225	0.8242
	Serifel_drench_03d	-0.4083333	1.206.771	16.14	-0.338	0.7394
	Serifel_drench_05d	0.1	1.079.369	16.14	0.093	0.9273
	Serifel_drench_08d	0.27833333	0.664071	75	0.419	0.6763
	Serifel_drench_10d	-0.20833333	0.664071	75	-0.314	0.7546
**EDS1**	Serifel_drench_07d	-0.3466667	1.404.289	20	-0.247	0.8075
	Serifel_drench_03d	-0.2966667	1.343.575	16.17	-0.221	0.828
	Serifel_drench_05d	-0.1766667	1.201.730	16.17	-0.147	0.8849
	Serifel_drench_08d	0.1833333	0.442983	75	0.414	0.6802
	Serifel_drench_10d	-0.945	0.442983	75	-2.133	0.0362
**ERF1**	Serifel_drench_07d	-1.24500000	0.917292	48	-1.357	0.181
	Serifel_drench_03d	0.1483333	0.578209	58	0.257	0.7984
	Serifel_drench_05d	-0.015	0.578209	58	-0.026	0.9794

	Serifel_drench_08d	-1.0066667	0.750829	75	-1.341	0.1841
	Serifel_drench_10d	-0.715	0.750829	75	-0.952	0.344
**LOXD**	**Serifel_drench_03d**	**0.95**	**0.509301**	**125**	**1.865**	**0.0645**
	Serifel_drench_05d	0.43833333	0.509301	125	0.861	0.3911
	Serifel_drench_07d	0.18	0.509301	125	0.353	0.7244
	Serifel_drench_08d	0.84083333	0.509301	125	1.651	0.1013
	**Serifel_drench_10d**	**1.29916667**	**0.509301**	**125**	**2.551**	**0.012**
**MYC2**	Serifel_drench_07d	-0.04333333	1.511.188	18	-0.029	0.9774
	Serifel_drench_03d	0.3966667	1.142.964	16.05	0.347	0.7331
	Serifel_drench_05d	0.7533333	1.022.298	16.05	0.737	0.4718
	Serifel_drench_08d	-0.375	0.299917	74	-1.250	0.2151
	Serifel_drench_10d	0.131666667	0.299917	74	0.439	0.6619
**NPR1**	Serifel_drench_07d	-1.153333	1.078.301	47	-1.070	0.2903
	Serifel_drench_03d	0.145	0.712392	58	0.204	0.8394
	Serifel_drench_05d	0.5683333	0.712392	58	0.798	0.4283
	Serifel_drench_08d	0.3933333	0.544429	80	0.722	0.4721
	Serifel_drench_10d	-0.7116667	0.544429	80	-1.307	0.1949
**PR1b1**	**Serifel_drench_03d**	**1.2558333**	**0.598325**	**125**	**2.099**	**0.0378**
	Serifel_drench_05d	0.0925	0.598325	125	0.155	0.8774
	Serifel_drench_07d	0.5291667	0.598325	125	0.884	0.3782
	Serifel_drench_08d	-1.2866667	0.598325	125	-2.150	0.0334
	Serifel_drench_10d	0.5216667	0.598325	125	0.872	0.3849
**Pti5**	Serifel_drench_07d	0.9733333	1.617.983	16.98	0.602	0.5554
	Serifel_drench_03d	1.1633333	1.418.743	16.18	0.82	0.4241
	Serifel_drench_05d	-0.1433333	1.268.963	16.18	-0.113	0.9115
	Serifel_drench_08d	-1.6683333	0.561781	75	-2.970	0.004
	Serifel_drench_10d	-0.25	0.561781	75	-0.445	0.6576

Supplementary Figure S3-comparisons of all treatments against control (water)

Gene	Treatment	estimate (Log2FC)	SE	df	t.ratio	p.value
**EXLB1**	Serifel_1st.spray_00h	-0.25833333	0.581898	70.74	-0.444	0.6584
	Serifel_1st.spray_01d	0.96833333	0.581898	70.74	1.664	0.1005
	Serifel_1st.spray_02d	0.26833333	0.581898	70.74	0.461	0.6461
	Serifel_1st.spray_03d	-0.54333333	0.581898	70.74	-0.934	0.3536
	Serifel_1st.spray_07d	-0.52833333	0.581898	70.74	-0.908	0.367
	**Serifel_1st.spray_08d**	**-1.78679674**	**0.628114**	**96.03**	**-2.845**	**0.0054**
	Serifel_1st.spray_10d	-0.93166667	0.581898	70.74	-1.601	0.1138
**FAD2**	Serifel_1st.spray_00h	0.26	0.642932	44.98	0.404	0.6878
	**Serifel_1st.spray_01d**	**1.26666667**	**0.642932**	**44.98**	**1.970**	**0.055**
	Serifel_1st.spray_02d	-0.25833333	0.642932	44.98	-0.402	0.6897
	Serifel_1st.spray_03d	-0.59	0.642932	44.98	-0.918	0.3637
	Serifel_1st.spray_07d	1.04833333	0.642932	44.98	1.631	0.11
	**Serifel_1st.spray_08d**	**2.57333333**	**0.642932**	**44.98**	**4.002**	**0.0002**

	Serifel_1st.spray_10d	1.20666667	0.642932	44.98	1.877	0.067
**GH3.8**	Serifel_1st.spray_00h	0.54166667	0.474233	177.36	1.142	0.2549
	Serifel_1st.spray_01d	0.88	0.474233	177.36	1.856	0.0652
	Serifel_1st.spray_02d	0.72666667	0.474233	177.36	1.532	0.1272
	Serifel_1st.spray_03d	0.58333333	0.474233	177.36	1.230	0.2203
	Serifel_1st.spray_07d	0.36166667	0.474233	177.36	0.763	0.4467
	Serifel_1st.spray_08d	0.08833333	0.474233	177.36	0.186	0.8524
	Serifel_1st.spray_10d	-0.17833333	0.474233	177.36	-0.376	0.7073
**ParA**	**Serifel_1st.spray_00h**	**1.25666667**	**0.604496**	**78.66**	**2.079**	**0.0409**
	**Serifel_1st.spray_01d**	**1.99333333**	**0.604496**	**78.66**	**3.298**	**0.0015**
	Serifel_1st.spray_02d	-0.205	0.604496	78.66	-0.339	0.7354
	Serifel_1st.spray_03d	-1.00833333	0.604496	78.66	-1.668	0.0993
	**Serifel_1st.spray_07d**	**1.19833333**	**0.604496**	**78.66**	**1.982**	**0.0509**
	**Serifel_1st.spray_08d**	**1.85333333**	**0.604496**	**78.66**	**3.066**	**0.003**
	Serifel_1st.spray_10d	0.06	0.604496	78.66	0.099	0.9212
**PP2C**	**Serifel_1st.spray_00h**	**-1.62833333**	**0.537995**	**112**	**-3.027**	**0.0031**
	Serifel_1st.spray_01d	0.31666667	0.537995	112	0.589	0.5573
	Serifel_1st.spray_02d	-2.21166667	0.537995	112	-4.111	0.0001
	**Serifel_1st.spray_03d**	**1.89666667**	**0.537995**	**112**	**3.525**	**0.0006**
	**Serifel_1st.spray_07d**	**1.10000000**	**0.537995**	**112**	**2.045**	**0.0432**
	Serifel_1st.spray_08d	0.15166667	0.537995	112	0.282	0.7785
	**Serifel_1st.spray_10d**	**-2.44833333**	**0.537995**	**112**	**-4.551**	**<.0001**

Supplementary Table S2. Contrasts and significance levels of treatments in gene expression analysis of the selected defense-related genes. Comparisons in boldface are discussed in main text.
